# Supplementary material for: A new species of the archaic primate Zanycteris from the late Paleocene of western Colorado and the phylogenetic position of the family Picrodontidae
Source: PeerJ. 2013 Oct 29;1:e191. doi: 10.7717/peerj.191 (PMC3817582; doi:10.7717/peerj.191)
Supplement: Supplemental Information 1 — List of characters used in phylogenetic analysis. [file peerj-01-191-s001.rtf]

Character List used in Phylogenetic Analysis 
(upper case letters indicate upper teeth, lower case letters indicate lower teeth)
Characters 15 through 111 based on Silcox, 2001
1. Large winged or rimmed postcingulum on M3.
	(Present=1, Absent=0) 
2. Square basin formed by expansion of postcingulum on M2.
	(Present=1, Absent=0)
3. Expansion of the postcingulum on M2.
	(Present=1, Absent=0) 
4. M1 is twice the size of M2 .
	(Present=1, Absent=0)
5.  Postprotocrista on M2  .
	(Present=0, Absent=1)
6. Flared parastyle on M2. 
	(Present=0, Absent=1)
7. M1 postprotocrista small or incomplete  .
	(Present=1, Absent=0)
8. M1 with a broad trigon basin, and precingulum lip.
	(Present=1, Absent=0)
9.  Cup or concave shaped M2 (with deep trigon basin).
	(Present=1, Absent=0)
10. The protocone is smaller on the edge of the anterior-lingual margin of the M2 .
	(Present=1, Absent=0)
11. A broad rounded lingual edge of the M2 formed by the postprotocingulum .
	(Present=1, Absent=0)   
12. Elongated postmetacrista on the M1.
	(Present=0, Absent=1)
13. Broad trigon basin with grooved crenulations. 
	(Present=1, Absent=0)
14. Centrocrista is straight, with no groove in the transverse direction.
	(Present=1, Absent=0)
15. Presence of i3.
	(Present=0, Absent=1) 
16. Presence of i2.
	(Present=0, Absent=1, Present and enlarged compared to i1 =2)
17. Presence of I3.
	(Present=0, Absent=1)
18. Presence of I2.
	(Present=0, Absent=1)
19. Size of i1.
	(Comparable to other incisors/premolars=0, Hypertrophied=1)
20. Orientation of i1.
	(Essentially vertical=0, Procumbent-horizontal=1)
21. Form of i1.
	(Simple, not laterally compressed=0, Laterally compressed with no broad flattened dorsal surface =1,  Similar to 1, but with a flattened dorsal surface=2, Similar to 2, but with rotated medially=3, Multicuspated or comb-like=4)
22. Presence of a margoconid on i1.
	(Absent=0, Present=1)
23. Enlargement of I1.
(Not markedly enlarged relative to other incisors or premolars =0, Enlarged=1)
24. Morphology of I1.
(No accessory cuspules=0, Posterocone present, but no apical cuspules=1, Posterocone and small cuspules developed around the tip with no strong apical division =2, Strong apical division into an anterocone and laterocone with a posterocone=3)
25. Relative size of the lower canine.
	(Larger than adjacent teeth=0, Smaller than adjacent teeth=1, Absent=2) 
26. Number of upper canine roots.
	(Single rooted=0, Double rooted=1, Absent=2)
27. Presence of p1.
	(Present=0, Absent=1)
28. Number of alveoli for p2.
	(Two alveoli=0, One alveolus=1, Tooth absent=2)  
29. Number of roots for p3.
	(Two roots=0, One root=1, Tooth absent=2)
30. Presence of P1.
	(Present=0, Absent=1)
31. Number of roots for P2.
	(Double rooted=0, Single rooted=1, Absent=2, Triple rooted=3)
32. Number of roots for P3 .
(Double rooted=0, Triple rooted=1, Absent=2)
33. Mesiodistal length of p4.
	(Slightly smaller than M1=0, Subequal=1, Larger than M1=2, Much smaller than M1=3)
34. Morphology of the p4 talonid.
	(Basined =0, Not basined, reduced=1, Not basined, expanded=2, Not basined, flap-like=3)
35. Number of p4 talonid cusps.
	(Two well defined=0, All poorly defined=1, One single distinct cusp=2, Three well defined cusps=3)  
36. Position of the p4 cristid oblique.
(Joins postvallid near midline of tooth or more lingually=0, Joins postvallid near buccal margin of trigonid=1, Absent=2)
37. Morphology of the hypoflexid on p4.
	(Distinct and deep=0, Not distinct and shallow=1)  
38. Presence of a mesoconid on p4.
	(Absent=0, Present (or variable)=1)
39. p4 paraconid presence.
(Paraconid distinct, cuspate=0, Cusp indistinct, but paracristid present, not markedly enlongated =1, paraconid and paracristid absent or weak=2, Paracristid elongate with or without a distinct cusp=3)
40. Presence of a p4 metaconid.
	(Absent=0, Present=1) 
41. Addition of apical cusps to p4 beyond the paraconid and metaconid.
(None=0, One neomorphic cusp (4 cusps in total)=1, Two neomorphic cusps (5 cusps in total)=2, Three or more neomorphic cusps (6 or more cusps in total)=3)
42. Morphology of the crest between the protoconid and cristid oblique on p4.
(Crest not continuous from postvallid to cristid obliqua=0, Crest present and continuous=1, No comparable crest on postvallid=2)
43. Presence of exodaenodont lobes on p4.
(Tooth not exodaenodont at all=0, Little lobe development=1, Distal lobe developed more than mesial =2, Both distal and mesial lobes strongly developed=3)
44. Presence of exodaenodont lobes on m1.
(Tooth not exodaenodont at all=0, Little lobe development=1, Distal lobe developed more than mesial=2, Both distal and mesial lobes strongly developed=3)
45. Presence of a protocone on P3.
	(Absent=0, Present=1)
46. Presence of conules on P3.
	(Absent=0, One or two present=1, Tooth Absent=2)
47. Presence of a preparaconule crista on P3.
	(Absent=0, Present=1, Tooth Absent=2)
48. Presence of conules on P4.
	(Absent=0, Large conule present=1, Small paraconule present=2, Both conules strong=3)
49. Presence of an intermediate ridge on P4.
	(Absent=0, Present=1)
50. Presence of an indentation on P3 postcingulum.
	(Absent=0, Present (with or without a cuspule)=1, Tooth is absent=2)
51. Presence of a P3 metacone.
	(Absent=0, Present=1, Tooth is absent=2)
52. Presence of a P3 metastyle.
	(Absent=0, Present=1, Tooth is absent=2)
53. Presence of a P4 metacone.
	(Absent =0, Present=1)
54. Presence of a P4 metastyle.
	(Absent=0, Present=1) 
55. Presence of a P3 parastyle.
	(Absent=0, Present=1, Tooth is absent=2)
56. Presence of a P4 parastyle.
	(Absent=0, Present=1)
57. Presence of an additional “preparaconal” cusp on P4.
	(Absent=0, Present between parastyle and paracone=1)
58. Presence of a hypocone on P3.
	(Absent=0, Present, at least incipiently=1, Tooth is absent=2) 
59. Presence of a hypocone on P4.
	(Absent=0, Present=1)
60. Presence of a pericone on P4.
	(Absent=0, Present, at least incipiently=1)
61. Shape of P3 (buccal length/lingual length).
	(Less than 1.7=0, Between 1.8-2.0=1, Greater than 2.0=2, Tooth is absent=2)
62. Shape of P4 (buccal length/lingual length).
(Less than 1.8=0, Greater than 1.8=2)
63. Relative size of P3 compared to P4.
	(Based on ln(buccal lengthXwidthP3/ln(buccal lengthXwidthP4)
	(Between 0.6 and 1.2=0, Less than 0.6=1, Greater than 1.2=2)
64. Relative size of P4 compared to M1.
	(Based on ln(buccal lengthXwidthP4/ln(buccal lengthXwidthM1)
	(Between 0.90 to 0.98=0, Greater than 0.98=1, Less than 0.90=2)
65. Presence of a postprotocingulum on P4.
	(Absent=0, Present=1)
66. Presence of a preprotocrista on P4.
	(Present=0, Absent=1)  
67. Presence of a postprotocrista on P4.
	(Present=0, Absent=1)
68. Morphology of the P4 parastylar lobe.
	(Large and projecting=0, Small and not projectiong=1)
69. Shape of the P4 protocone lobe.
	(Shorter mesiodistally than wide=0, Equally long and wide=1)  
70. Acuteness of P4 cusps, when unworn.
	(Acute=0, Bulbous=1)
71. Distinctiveness of the molar hypoflexids.
	(Distinct, invaginated=0, Not distinct=1)
72. Molar trigonid height on M1-M2.
	(Tall relative to talonid=0, Similar height to talonid=1) 
73. Molars high-crowned.
	(High crowned =0, Moderate =1, Low crowned=2)  
74. Presence of buccal cingulids on the lower molars.
(Anterobuccal “precingulid” only=0, Separate anterior and posterior cingulids=1, Continuous buccal cingulid=2, Absent, no buccal cingulid or “precingulid”=3)
75. Length of molar trigonids.
(Trigonids become less mesiodistally compressed from m1-3=0, No change=1, Trigonids become more mesiodistally compressed from m1-3)
76. Height of the lower molar paraconids.
	(Lower than metaconid=0, Subequal to or taller than the metaconid=1)
77. Distinctiveness of the paraconid on m1.
	(Indistinct from paracristid=0, Distinct from paracristid=1)
78. Presence of paraconids on m2.
	(Comparably distinct to the paraconid on m1=0, Less distinct than the paraconid on m1=1)
79. Presence of lower molar mesoconids.
	(Weak-absent=0, Present (if variably) on some molars=1)
80. Molar enamel roughness.
	(Smooth=0, Crenulated=1, Papillated=2)  
81. Relative positions of the m1-2 hypoconulid and entoconid.
	(Unpaired=0, Paired=1, Twinned=2)  
82. Position of m1 and m2 hypoconulid relative to the central axis of the tooth.
(Hypoconulid centrally placed or shifted lingually towards the entoconid=0, Hypoconulid shifted bucally=1)
83. Presence of an entoconid-hypoconulid notch on m1 and m2.
	(Absent=0, Notch present=1)
84. Presence of a hypoconulid-hypoconid notch on m1 and m2.
	(Absent=0, Notch present=1)
85. Presence of mesial inflection of the molar trigonids.
	(Absent=0, Weak=1, Pronounced=2)
86. The basal breadth of the m1 and m2 trigonids.
	(Not swollen at the base=0, Swollen basally=1)
87. Breadth of the m1 and m2 talonids near the apices of the cusps.
	(Narrower than trigonid=0, Wider than trigonid=1)
88. The breadth of the m3 talonids.
	(Much narrower than trigonid=0, Similar in breadth to trigonid or wider=1)
89. Size of the m3 hypoconulid.
(Similar to that cusp on m1 and m2=0, Larger than on m1 and m2, but not developed into a lobe=1, Developed into a lobe=2)  
90. Relative height of the molar talonid cusps.
	(Hypoconid taller than entoconid=0, Entoconid taller=1).
91. Relative height of the protoconid and metaconid on m1.
	(Protoconid higher=0, Subequal=1, Metaconid higher=2)
92. Presence of curving molar paracristids.
	(Absent=0, Present=1)
93. Morphology of the protoconid-metaconid notch on the lower molars.
	(Strong and sharp=0, More rounded=1, Fold of enamel bridges notch on the lower molars=2)
94. Presence of a stepped postvallid on m1.
	(Absent=0, Present=1)
95. Acuteness of upper and/or lower molar cusps (unworn).
	(Relatively acute or sharp=0, Blunter=1)  
96. Relative size of M3.
	(ln(buccal length X width M3)/ln(buccal length X width m1))
	(Less than 0.9=0, Greater than 0.9=1)
97. Relative length of m3.
	(m3 is smaller than m2=0, m3 is larger than m2=1)
98. Morphology of the molar stylar shelf.
	(Broad=0, Narrow (buccal cingulum only) or absent =1)
99. Presence of a postprotocingulum on M1 or M2.
	(Absent=0, Weak=1, Pronounced=2)  
100. Position of the conules on M1 and M2.
	(Central or closer to protocone than to paracone/metacone=0, Apressed to paracone/metacone=1)
101. Presence of conules on M1 and M2.
	(Both conules present=0, Metqaconule absent=1, Both absent=2)
102. Relative sizes of metacone and paracone on M1 and M2.
	(Paracone larger than metacone=0, Cusps are subequal=1, Metacone larger than paracone=2)
103. Presence of a hypocone on M1 and M2.
(Absent=0, Present (“true hypocone”, coming off the cingulum)=1, Present (“pseudohypocone”, budding off the protocone)=2)
104. Position of the M1 and M2 protocone.
	(Skewed mesiobuccally=0, Centrally located on tooth =1) 
105. Presence of a precingulum on M1 and M2.
(Present, doesn't connect to postcingulum=0, Present, connects to postcingulum (can be variable)=1, Absent=2)
106. Continuity of post- and metacingula.
	(Not continuous =0, Continuous =1)
107. Separateness of pre- and paracingula on M1 and M2.
	(Not continuous= 0, Continuous =1, No paracingulum=2)
108. Morphology of the centrocrista on the upper molars.
	(Moderate=0, Strong and straight=1, Absent or very weak=2, Strong and V-shaped=3)
109. Morphology of the parastylar lobe on M1.
	(Projecting beyond the plane of the mesiolingual corner of the tooth, Not projecting=1)
110. Presence of none or more mesostyle(s) on the upper molars.
	(Absent=0, One or more present=1)  
111. Orientation of the preparacrista on M1 and M2.
	(Angled buccally=0, Straight=1)
112. I2 is enlarged in comparison to other incisors.
	(Small I2=0, Enlarged I2=1)  
113. P2 overlays lower incisor.
	(Absent=0, Present=1) 
