# Supplementary material for: A new species of the archaic primate Zanycteris from the late Paleocene of western Colorado and the phylogenetic position of the family Picrodontidae
Source: PeerJ. 2013 Oct 29;1:e191. doi: 10.7717/peerj.191 (PMC3817582; doi:10.7717/peerj.191)
Supplement: Supplemental Information 2 [file peerj-01-191-s002.rtf]

#NEXUS

begin data;
dimensions ntax=58 nchar=113;
format datatype = standard gap = - missing =?;
matrix

Paradectes_var._sp.   00000000000000000000000000000000000000000000000000????????????????????0003101000200000010100000000000001200300000
Cimolestes_var._sp.   00000000000000?????????????00???312000000000??????????????????????????0003101000101100001010000010000001200300000
Leptacodon_var._sp.   00000000000000000000000000000000010000200000000000000000000000000000000003101000100000000010000000000010000300000
Labidolemur_kayi   00000000000000111011101122101112312000200000022002221120020030110000000003101000000010000000000000000010000000011
Labidolemur_major   ?0000?00000000111011101122101112312000000000022002221120020030000000000003000000000000000000000000000010000000011
Labidolemur_serus   00000000000000111011101122101112312000000000022002221120020030000000000003000000000000000000000000000010000000011
Labidolemur_cf._kayi   00000000000000111011101122101112312000000000022002221120020030000000000003000000000000000000000000000010000000011
Planetetherium_mirabile   ?0000100000100??????????????????103100010211100300100000001011000001100102100000201100111120000110000110000311000
Elpidophorus_elegans   00000100000100?0001140?????10?311031000102111003001000000010?1?00001100102100000201100111121?00010000110000311000
Plagiomene_multicuspis   0000010000010000001140??0?010031103100010212110300110100000011010001100102201000201100111120000110000100010311000
Horolodectes_sunae   0000010000010000??000?0?00010031111200210212100000101011000011110001000103201000201100111010001110000100000310000
Worlandia_inusitata   0000010000010010??10????00010031103001010212110300100011000011010001010102201000201101111010101110000100000310000
Purgatorius_unio   000001000000000011??0???????????000000(0 1)00100??????????????????????????0000(0 1)0(0 1)000000010001001000110(0 1)00000000000000
Purgatorius_coracis   000001000000000011110?1200000??1000000000000100000100010000020001000000000(0 1)0(0 1)000000010101001000010(0 1)00001000000000
Berruvius_gingerichi   ?0000100000000?0??1110??1??{0 1}0???000000110000???20???00?00?00????0010000000100000000010101101000101110010000010000
Navajovius_kohlhaasae   0000010000000011??1130??1111010000000011000000000000101000100010101000001010100010011010100100010111001000001?100
Niptomomys_doreenae   ?000010000010010101130112111110001110010010000000000101000000010101001012(0 3)201101101110111101101111010000000010100
'Arctodontomys simplicidens/wilsoni'   0000010000010011??1130??2?111??100000010010000000000(0 1)?1??00?01121010000002201011101110111101000111110010011010100
Micromomys_fremdi   0000010000010011??11????1?10010120(0 1)000300220001000000010000020000000100000000000100110101101000011001000000000000
Chalicomomys_antelucanus   000001000000001???11????1?110??12000003002101002000000100000200000001000101010001001101??101001011210000000010100
Tinimomys_graybulliensis   00000100000100111011101211120101200000300220100200000010000020(0 1)?0000000010101010100110111101001111(0 1)100101(0 1)0010100
Dryomomys_szalayi   0000010000010011?0111011??020101201000300220100200000010000020100000000010101010100110111101001111(0 1)100101(0 1)0010100
Palaechthon_alticuspis   0010010000000010??11????1?110?01000(0 1)00(0 1)(0 1)010000020000001000000100101010000(0 2)2010100000101120210001112100000(0 1)(0 1)01?000
Anasazia_williamsoni   00100100000000????11??120??10????0000000010???????????????????????????00022010100000101120110001102100000000?0000
Torrejonia_wilsoni   00100100000100?????1????????0???000110(0 1)(0 1)0100???20???00?00?00?1??101011000(0 2)201011000110111001001?11210100000000000
Phoxomylus_puncticuspis   ?0100100000100????????????????????????????????????????????????????????????????????????????????????210100000000000
Plesiolestes_nacimienti   0010010001110000??11????00110101000000??0?0000000010001000000112100010000(0 2)201000000110112011000111210000000000000
Plesiolestes_problematicus   0000010000010010??1120??00110101103101(0 1)1010000020010000000002100101010000(0 2)201010100110112011100011210000000000000
Talpohenach_torrejonius   00100100001100???????????0???101????????????10020010000000002102101010???????0??0?????????????00?1210100011010000
Paromomys_maturus   0010010000110010??11????0?10010100000010010000000000(0 1)01(0 1)000001011010100112201101000020112111200111211000010010000
Phenacolemur_pagei   1110010001110011??11101321122120100110200100000000100010000011001010100113201101000020112001200011211000010010000
Phenacolemur_praecox   1110010001110011??1120132?122??1100110200100000200100010000011001010100113201101000020112021200011211000010010000
Phenacolemur_jepseni   1110010001110011??111013211221201001102002000000001000100000????1010100113201100000020112001200011211000010010000
Ignacius_clarkforkensis   011001000111001110111013201221213001102001000002001000(0 1)0000001021010100113201100000020112021200011211000010010000
Acidomomys_hebeticus   011001000111001010101013211201?0???????????00002001000(0 1)0000001021010100113201100000020112021200011211000010010000
Ignacius_frugivorus   011001000111001110111013201221113001102001000002001000(0 1)0000001021010100113201100000020112021200011211000010010000
Draconodus_apertus   ?1111000111000??????????????????????????????????????????????????????????2??????2??????????????0??02?210000000?000
Picrodus_calgariensis   0111101111100110??1120??22111???311210200202???00???10?10?00?1??011110102(0 3)(1 2)01002?01120110?010001002?2100002110000
Picrodus_canpacius   0111101111100110??1120??22111???311210200202???00???10?10?00?1??011110102(0 3)(1 2)01002?01120110?010001002?2100002110000
Picrodus_lepidus   0111101111100110??1120??22111???311210200202???00???10?10?00?1??011110102(0 3)(1 2)01002?01120110?010001002?2100002110000
Picrodus_silberlingi   ?111101111100110??1120??22111???311210200202???00???10?10?00?1??011110102(0 3)(1 2)01002?01120110?010001002?2100002110000
Zanycteris_paleocenus   01111000111011??????????0????100????????0??????????????????????????????????????2??????????01??01?02?2100000100000
Zanycteris_honeyi   ?1111010111011??????????0????100????????0??????????????????????????????????????2??????????01??01??2?2100000100000
Pronothodectes_matthewi   0000010000010010??11211310110101311200200200110100100011000011021011110010211010000011112101111011210001111210?00
Pronothodectes_gaoi   0010010000010010??1121??1?110??1311200200200110100100000000011021011110110211000000011112111111?11210001010210100
Nannodectes_gazini   00000100000100111011211320120121311200200200110100100000000000021011110010211001000011112001111011210001010210100
Plesiadapis_fodinatus   00000100000100111011211320120101311200200200110100100000000001021011010010211011000011112001111011210001010211100
Plesiadapis_dubius   00000100000100111011211322120101311200200200110100100000000001021011010010211011000011112001111011210001010211100
Plesiadapis_cookei   00000100000100111011211322120??13112102002001000000000110000?1?2101111011(0 2)2110010000111120111110112100(0 1)1111211100
Elphidotarsius_wightoni   0000010000010010??112113101200112122000111111111111100001000110110111000002110000001111121010100112100110000101?0
Elphidotarius_russelli   0000010000010010??11??131?121??121220001(1 2)1331101111101001111110111111000002110000001111121010100112100110000101?0
Carpolestes_simpsoni   00000100000100100011201312122111212200014133111111110100111111011111100000211000000?111121010100112100110000101?0
Carpolestes_dubius   000001000001001000112013021210112122000141331111111101001111110111111000002110000001111121010100112100110000101?0
Carpodaptes_hazelae   0000010000010010001120130212101121220001(2 3)13311(0 1)1111101001111110111111000002110001001111121010100112100110000101?0
Carpodaptes_cygneus   000001000001001000112013021210112122000141331111111101001111110111111000002110000001111121010100112100110000101?0
Carpodaptes_stonley   0000010000010010001120130212101121220001(2 3)13311(0 1)1111101101111110111111000002110000001111121010100112100110000101?0
Carpomegodon_jepseni   00000100000100100011201302121011212200014133111111110100111111011111100000211000000?111121010100112100110000001?0
Saxonella_naylori   0000010000010011??1110??2?12010101221010020011111010000100002121111100000320(0 1)0000000111??1010000112100010010101?0

;
end;

BEGIN SETS;
	CHARPARTITION * matrices = Character_Matrix : 1-113 ;
	charset Character_Matrix = 1-113;
END;

begin mrbayes;
	charset Character_Matrix = 1-113;
	partition matrices = 1: Character_Matrix;
	set partition = matrices;
	unlink statefreq=(all) revmat=(all) shape=(all) pinvar=(all); 
	prset applyto=(all) ratepr=variable;
	mcmcp ngen= 10000000 relburnin=yes burninfrac=0.5 printfreq=1000  samplefreq=1000 nchains=4 savebrlens=yes;
	mcmc;
end;
